# The Sweet-Side of Leukocytes: Galectins as Master Regulators of Neutrophil Function

**DOI:** 10.3389/fimmu.2019.01762

**Published:** 2019-08-07

**Authors:** Brian S. Robinson, Connie M. Arthur, Birk Evavold, Ethan Roback, Nourine A. Kamili, Caleb S. Stowell, Mary L. Vallecillo-Zúniga, Pam M. Van Ry, Marcelo Dias-Baruffi, Richard D. Cummings, Sean R. Stowell

**Affiliations:** ^1^Department of Laboratory Medicine and Pathology, Center for Transfusion Medicine and Cellular Therapies, Emory University School of Medicine, Atlanta, GA, United States; ^2^Department of Biochemistry, Brigham Young University, Provo, UT, United States; ^3^Department of Clinical Analyses, Toxicology and Food Sciences, School of Pharmaceutical Sciences of Ribeirao Preto, University of São Paulo, São Paulo, Brazil; ^4^Department of Surgery, Beth Israel Deaconess Medical Center, Harvard Medical School, Boston, MA, United States

**Keywords:** neutrophil, galectin, glycoscience, inflammation, glycans

## Abstract

Among responders to microbial invasion, neutrophils represent one of the earliest and perhaps most important factors that contribute to initial host defense. Effective neutrophil immunity requires their rapid mobilization to the site of infection, which requires efficient extravasation, activation, chemotaxis, phagocytosis, and eventual killing of potential microbial pathogens. Following pathogen elimination, neutrophils must be eliminated to prevent additional host injury and subsequent exacerbation of the inflammatory response. Galectins, expressed in nearly every tissue and regulated by unique sensitivity to oxidative and proteolytic inactivation, appear to influence nearly every aspect of neutrophil function. In this review, we will examine the impact of galectins on neutrophils, with a particular focus on the unique biochemical traits that allow galectin family members to spatially and temporally regulate neutrophil function.

## Introduction

A hallmark of effective immunity is the ability to rapidly recognize and respond to invading pathogens while avoiding potential injury to surrounding host tissue. This is especially important during the initial recruitment of neutrophils, one of the earliest and most effective responders to microbial infection (1). Neutrophils express a wide variety of potent antimicrobials, including degradative enzymes and highly reactive free radicals that can neutralize and ultimately kill many different invading pathogens (2–5). Although neutrophils can cooperate with antibodies to focus their effector function toward individual microbes, during the primary exposure to a given microbe, neutrophils rely on less specific mechanisms to recognize and respond to infection (6). Poorly controlled neutrophil infiltration and activation can result in significant tissue injury (3, 7–9). In contrast, inadequate neutrophil mobilization and activation can prevent rapid microbial eradication. In order to effectively defend against invading microbes, while limiting host injury, the localization, activation and eventual removal of neutrophils must be tightly regulated to efficiently eliminate potential pathogens while avoiding additional tissue damage and increased organ dysfunction (3, 7–11).

The importance of appropriately governing early immune effectors is especially apparent in disease states in which neutrophil regulation is compromised. Genetic disease that impair neutrophil recruitment to sites of infection or reduce neutrophil effector activity leave patients prone to infectious disease (12–14). Similarly, patients who are neutropenic secondary to bone marrow dysfunction or other etiologies are particularly prone to life threatening infection (15). In contrast, excessive neutrophil recruitment and activation often contributes to the pathogenesis of some forms of inflammatory bowel disease, reperfusion injury or unabated infection (7–9). Thus, while neutrophils provide a critical defense mechanism against possible infection, the appropriate regulation and eventual elimination of these early immune effectors is critical if host defense is to be achieved while avoiding additional host injury (10, 11).

While a variety of factors regulate neutrophil recruitment, activation and eventual removal, many studies have demonstrated that a series of carbohydrate binding proteins (CBPs) called galectins play a key role in this process. Galectin family members recognize highly modifiable cell surface carbohydrates to facilitate neutrophil extravasation, activation, microbial killing, and eventual turnover. While there have been many excellent reviews detailing the regulatory roles of galectins in general on immune activity and function (16–19), in this review we will specifically examine the role of galectins in regulating neutrophil function. We will focus on the impact of unique aspects of galectin biochemistry that may contribute to the ability of this CBP family to influence various aspects of neutrophil function.

## Galectins

Shortly after the identification of the first mammalian CBP, the Ashwell-Morell receptor, now known to govern platelet turnover and production (20–23), several studies sought to determine whether vertebrates possess other CBPs. In 1975, based on the ability of the Ashwell-Morell receptor to recognize terminal galactose residues, Teichberg and colleagues used a similar approach to isolate electrolectin, the ortholog of galectin-1 from the electric organ of the electric eel (24). While other investigators initially failed, the ability of Teichberg and colleagues to isolate and subsequently characterize the first galectin resulted from the inclusion of reducing agents in their isolation buffers (24, 25). Failure to include reducing agents in isolation buffers allowed electrolectin to undergo oxidation, rendering the protein inactive with respect to its carbohydrate binding activity (26, 27). Following the initial isolation and characterization of electrolectin, subsequent studies demonstrated that several other members of the galectin family were also sensitive to oxidative inactivation (28–33). In doing so, these early studies uncovered one of the most distinguishing, yet often overlooked features of galectins, their sensitivity to oxidative inactivation. Given the unique requirement of early galectins for reduced thiols, galectins were initially referred to as S-type lectins to differentiate them from subsequently discovered vertebrate CBPs that required Ca^2+^ to recognize cognate ligand, coined C-type lectins (34).

**Figure 1:**
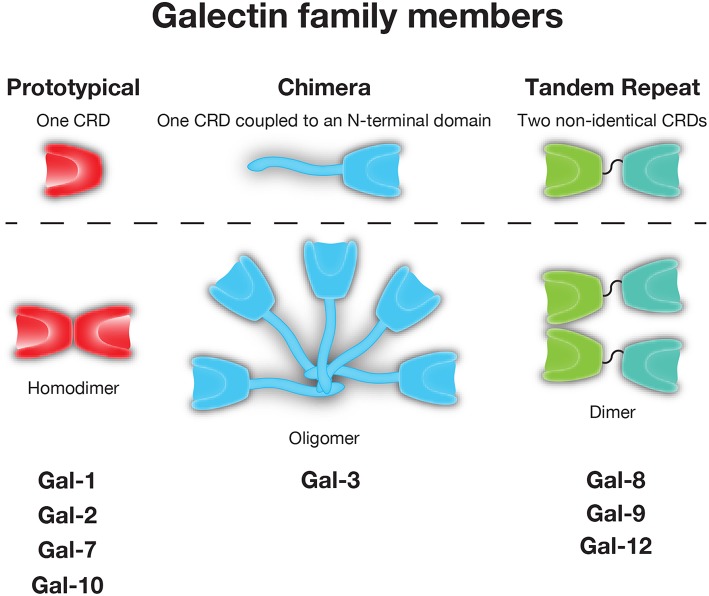
The galectin family. Galectins are divided into prototypical, chimeric, and tandem repeat subfamilies. Representative members of each family are shown.

As not all galectins require reduced sulfhydryls to maintain carbohydrate binding activity (35), yet each appeared to share the ability to recognize β-galactose containing glycans, these CBPs later became distinguished by the name galectin (36). While galectins are unified by their conserved binding affinity for β-galactoside residues, other galactose binding proteins have been described in metazoans. As a result, galectins have been distinguished from these CBPs by the lack of calcium-dependence in glycan binding that is observed in C-type lectins, the presence of a conserved carbohydrate recognition domain (CRD) with highly conserved amino acids required for glycan binding and secretion through a unconventional secretory pathway, which has only recently begun to be characterized (37). In all, over 15 galectins have been described in vertebrates. Typically categorized based on their tertiary and quaternary structure, galectins are often placed into one of three groups: prototypical (e.g., Gal-1, -2, -7, -10), which form homodimers containing one CRD, tandem-repeat (e.g., Gal-4, -8, -9, and -12), which contain two CRD's in tandem joined by a linker region, and chimeric (e.g., Gal-3), which have an N-terminal tail that allows for oligomerization and/or unique protein interactions outside the Gal-3 CRD (38) (Figure 1).

## Galectins Regulate Neutrophil Activation

Given the soluble nature of galectins, coupled with their ability to recognize highly modifiable glycan structures, galectins have served as a unique substrate in the evolution of immune regulation. The implication that galectins could influence leukocyte biology, in particular neutrophils, was originally described in studies designed to define interactions between leukocytes and IgE. Previous studies had suggested that IgE could activate neutrophils. However, the mechanism whereby this occurred remained incompletely understood. Surprisingly, biochemical studies seeking to first define the receptor responsible for the impact of IgE on neutrophil function found that rather than expressing conventional IgE-receptors (including Fc epsilon RII/CD23), neutrophils exhibited elevated expression of the S-type lectin Mac-2/Epsilon-bp (i.e., galectin-3) (39), a protein which had previously been shown to bind IgE *in vitro*. Importantly, galectin-3 interactions with IgE on the neutrophil surface resulted in NADPH-oxidase activation and a respiratory burst; neutralizing antibodies against Gal-3 prevented this IgE-mediated effect on neutrophil activation, strongly suggesting that Gal-3 serves as the primary IgE receptor on the surface of neutrophils (40). Gal-3 may therefore regulate neutrophil sensitivity to IgE mediated activation following allergen exposure in at risk patients (41).

The ability of Gal-3 to regulate neutrophil activity through IgE engagement suggested that Gal-3 itself may influence neutrophil function. Subsequent studies demonstrated that Gal-3 can initiate neutrophil oxidative responses. In this setting, recombinant Gal-3 not only binds to neutrophils and stimulates superoxide production, but also directly activates neutrophils completely independent of IgE, in a carbohydrate- and dose-dependent manner (42). Gal-3 oligomerization of potential counterreceptors appears to be required for the induction of ROS production, as the C-terminal domain of Gal-3 (Gal-3C), which has been shown to be defective in oligomerization (43, 44), fails to similarly induce neutrophil ROS (45). Furthermore, antibodies that enhance Gal-3 oligomerization also appear to facilitate Gal-3-induced ROS production (46).

Gal-3 dependent activation of neutrophil NADPH-oxidation occurs preferentially on exudated, but not peripheral (e.g., quiescent) neutrophils, implicating a role for priming events in the sensitization of neutrophils to Gal-3 (47). Priming events that render neutrophils sensitive to Gal-3 are not limited to extravasation, but also include exposure to lipopolysaccharide or lipoarabinomannans from gram negative microbes or mycobacteria, respectively (48–50). Despite the ability of Gal-3 to recognize strain specific carbohydrate O antigen and the lipid A of some forms of LPS (51–53), LPS from a variety of gram negative microbial strains, including *Escherichia coli, Klebsiella pneumoniae*, and *Salmonella minnesota*, possess this priming activity (50), suggesting that this priming event does not likely reflect enhanced Gal-3 binding at the neutrophil surface through LPS carbohydrate engagement. Newcastle disease virus neuraminidase sensitizes neutrophils to Gal-3 (54). As Newcastle disease virus neuraminidase also sensitizes neutrophils to fMLP (54), increased sensitivity may not result from direct exposure of Gal-3 receptors, but instead may reflect general alterations in sensitivity of neutrophils to common activators. In contrast to the extrinsic impact of Gal-3 on neutrophil ROS production primed under various conditions, intrinsic neutrophil Gal-3 appears to attenuate ROS production following *Candida albicans* exposure (55).

In addition to the impact of exposure to distinct microbial products or even microbes themselves on Gal-3 regulation of neutrophil activity, different disease and developmental states may also influence neutrophil sensitivity to Gal-3-induced ROS production. For example, while neonatal immunity is thought to be developmentally immature and less responsive to activating stimuli (56), neutrophils isolated from cord blood are actually more sensitive to Gal-3-induced ROS production than peripheral blood neutrophils isolated from adults (57). Neutrophils isolated from patients with periodic fever, aphthous stomatitis, pharyngitis and cervical adenitis (PFAPA) syndrome also experience enhanced ROS response following Gal-3 exposure (58), suggesting that other inflammatory mediators may provide surrogate cues that also prime neutrophils to Gal-3.

In addition to inducing ROS, subsequent studies demonstrated that Gal-3 facilitates neutrophil activation, as evidenced by enhanced L-selectin shedding, increased CD11b expression and IL-8 secretion (59, 60). Gal-3-induced ROS also has consequences on neutrophil sensitivity to additional activators. In conjunction with myeloperoxidase, ROS induced by Gal-3 results in fMLP degradation, which in turn reverses the desensitization neutrophils can experience following exposure to a higher concentration of fMLP (61). As this allows neutrophils to become more sensitive to fMLP, Gal-3-induced ROS provides a positive feedback loop that occurs when both galectins and bacterial products are present. In this setting, Gal-3 may serve as a damage associated molecular pattern molecule that signals the presence of tissue injury in the setting of microbial invasion (62).

In contrast to facilitating additional activation in the setting of infection, primed neutrophils can cleave Gal-3 into products no longer capable of signaling neutrophil ROS production, suggesting a potential negative feedback loop on Gal-3-mediated neutrophil activation. Importantly, cleaved, but not intact, Gal-3, is also preferentially internalized, raising the possibility that this cleavage event may also enhance Gal-3 removal (60). While Gal-3C can serve as a dominant negative regulator of Gal-3 activity on neutrophils, Gal-3C not only fails to prevent Gal-3 engagement of neutrophil ligands, but actually augments Gal-3 binding (45), making it unclear how Gal-3C modulates Gal-3 signaling. As Gal-3C can be detected on the surface of neutrophils in circulation (45), and extravasation appears to impact cell surface galectin and galectin-ligand levels (63), internalization of Gal-3C may serve as an additional regulator of neutrophil sensitivity to Gal-3 exposure following extravasation.

Early studies also demonstrated that in addition to Gal-3, Gal-1 can induce neutrophil ROS production, with extravasated and not quiescent neutrophils likewise exhibiting the most sensitivity to Gal-1 (64). Subsequent studies confirmed that Gal-1 from a variety of sources could induce ROS production, lysosome release and generalized degranulation (65, 66). Similarly, Gal-8 also stimulates ROS production, signaling primarily through its C-terminal domain (67, 68), while Gal-9 causes neutrophil degranulation and ROS production through a Tim3-dependent pathway (69, 70), suggesting that regulation of neutrophil ROS may be a more generalized galectin phenomenon. Patients with alcohol-induced hepatitis experience elevated levels of Tim3, PD1, PD-L1, and Gal-9 with impaired neutrophil ROS production and phagocytosis, which are reversed with anti-Tim3 and PD1, likewise implicating at least a partial role for Gal-9 *in vivo* in this process (71).

## Galectin Regulation of Neutrophil Extravasation

In addition to regulating neutrophil NADPH-oxidase activity, galectins have also been implicated in regulating neutrophil extravasation. Early studies observed that injected Gal-1 could reduce phospholipase A2-induced neutrophil accumulation, a process that was inhibited by lactose and anti-Gal-1 antibodies (72). Injection of Gal-1 similarly impairs carrageenan-induced neutrophil extravasation into the peritoneal cavity (73, 74), while Gal-1 likewise attenuates neutrophil infiltration in the setting of ocular inflammation (75). While these reductions could also reflect Gal-1-mediated alterations in neutrophil chemotaxis, pre-incubation of neutrophils with Gal-1 inhibits neutrophil rolling on endothelial cells, while increased neutrophil rolling was noted *in vivo* in response to IL1β in Gal-1 KO mice (63, 76), suggesting a direct effect. However, Gal-1 can limit neutrophil infiltration and Th17 responses following corneal exposure to *Pseudomonas aeruginosa*, suggesting that reductions in Th17 cells may also contribute to decreases in neutrophil infiltration in some settings (77). Similarly, Gal-1 treatment in a model of OVA-induced conjunctivitis reduces both pro-inflammatory cytokine production and neutrophil numbers (78). In contrast, Gal-1 KO mice infected intratracheally with *Histoplasma capsulatum* exhibit an elevated neutrophil pulmonary accumulation that may reflect higher chemokines levels for neutrophils (79).

In contrast to Gal-1, Gal-3 mediates neutrophil adhesion to endothelial cells, suggesting that Gal-3 may positively regulate neutrophil extravasation (80, 81). Consistent with this, Gal-3 injection decreases neutrophil rolling, while increasing adhesion and emigration (82). Gal-3 KO neutrophils exhibit an impaired ability to role on WT endothelium, suggesting a neutrophil intrinsic role for Gal-3 (82). Experimental models of infection appear to confirm a role for Gal-3 in neutrophil extravasation in some settings. Gal-3 KO recipients experience reduced neutrophil broncheoalveolar lavage (BAL) numbers following *Streptococcus pneumoniae* pulmonary infection. Reductions in neutrophil accumulation correlate with increased *S. pneumoniae* burden, an outcome that can be partially reversed by intranasal delivery of recombinant Gal-3 (83). Gal-3 KO mice also exhibit impaired neutrophil recruitment following thioglycolate-induced peritonitis (84) and *Leishmania major* skin infection (85). Although Gal-3 can induce macrophages and other cells to secrete pro-inflammatory cytokines and chemokines (86, 87) and Gal-3 injection can increase IL-1β, TNFα, CCL2, CXCL1, and IL-6 (82), impaired neutrophil recruitment does not appear to reflect a lack of cytokine or chemokine production, as neutrophil mobilization defects observed in Gal-3 KO mice can occur in the presence of increased levels of KC, MIP2, IL-6, and TNFα (60, 83, 85). In contrast, inhibition of Gal-3 can result in reduced TNFα, KC, TGFβ, and MCP-1 levels and neutrophil accumulation, as observed in a pancreatitis model, suggesting that Gal-3 may similarly facilitate extravasation and possibly chemotaxis in this setting (88). Occasionally, reduced neutrophil extravasation may be beneficial to the host. In a model of *Francisella novicida* pulmonary infection, reduced inflammation and neutrophil extravasation in Gal-3 KOs actually correlated with enhanced survival despite no difference in CFU numbers (89).

While the above studies have highlighted a role for Gal-3 in facilitating neutrophil extravasation, several studies suggest that Gal-3 may negatively regulate neutrophil extravasation in certain settings. Gal-3 KO mice actually experience increased neutrophil accumulation and disease severity in several infectious disease models, including neurocysticercosis (90) and polymicrobial sepsis (91). Similarly, a higher number of neutrophils can actually be detected in the BAL of Gal-3 KOs following pulmonary *E. coli*, as opposed to *S. pneumoniae*, infection (83). In contrast, Gal-3 KO mice appear to initially have similar neutrophil numbers in a spinal cord injury model (92) and likewise fail to display significantly different numbers in the setting of dextran sulfate sodium (DSS)-induced colitis (86), suggesting that in some settings Gal-3 may have an redundant role or simply no role in neutrophil recruitment. Finally, in addition to Gal-1 and Gal-3, other galectins have shown an ability to potentially regulate neutrophil extravasation. Gal-8 can also mediate neutrophil adhesion to endothelial cells (93), although the consequences of this interactions *in vivo* remain incompletely studied.

## Galectins Regulate Neutrophil Chemotaxis and Phagocytosis

Early studies found that Gal-3 could promote neutrophil cationic-dependent and independent binding to laminin, and at high concentrations could facilitate fibronectin binding (94, 95), suggesting that once neutrophils extravasate, Gal-3 may facilitate attachment and chemotaxis along extracellular matrix (ECM) glycoproteins. Gal-1 also recognizes laminin, fibronectin and neutrophil ligands, although the potential ability of Gal-1 to tether neutrophils to these ECM glycoproteins was never formally tested in these early studies (96, 97). Gal-1 does inhibit neutrophil chemotaxis in response to IL-8 *in vitro* and similarly reduces neutrophil transmigration following IL-1β-induced peritonitis (63). As it is difficult to distinguish extravasation and chemotaxis *in vivo*, alterations in neutrophil accumulation in these models may reflect a role for Gal-1 on neutrophil extravasation, chemotaxis or both (63, 72–78). Different neutrophil responses to Gal-1 may also reflect Gal-1 concentration; lower concentrations of Gal-1 can produce directed neutrophil movement, while higher concentrations appear to induce random motion (98). Intriguingly, whereas Gal-3 promotes extravasation into inflamed tissues (82), Gal-3 can inhibit leukocyte migration in response to IL-8, C5a, and ATP (99).

In addition to Gal-1 and Gal-3, Gal-9 also regulates neutrophil chemotaxis. Following ischemic injury, Gal-9 KO mice experience increased neutrophil infiltration that is partially reversed following injection of recombinant Gal-9 (100). Injected Gal-9 also reduces neutrophil accumulation in a model of emphysema (101), ConA-induced hepatitis (102) and reperfusion liver injury (103). Gal-9 KOs also experience a reduced neutrophil response to *Francisella novicida* pulmonary infection (70). However, as Gal-9 treatment can also reduce IL-6, IL-1β, IFNγ, TNFα, KC, MIP2, GM-CSF, and MMP9 in various models (70, 100, 102–104), alterations in neutrophil accumulation may reflect modulation of neutrophils by regulating either extravasation, chemotaxis, cytokine, and chemokine secretion or a combination of these events. Consistent with this, Gal-9 induces IL-8 production through engagement of Tim-3 on bronchial epithelial cells, resulting in neutrophil recruitment (105). Gal-9 may also regulate neutrophil infiltration by signaling changes in Treg activity, Th17 responses or T cell turnover (104, 106–108). Consistent with a more indirect role for Gal-9 in modulating neutrophil chemotaxis, early studies suggested that Gal-9, originally coined eotaxin, exhibits chemotactic activity toward eosinophils, yet fails to alter neutrophil, monocyte or lymphocyte chemotaxis (109, 110).

In addition to modulating neutrophil extravasation, chemotaxis and activation, galectins may also facilitate neutrophil phagocytosis. Gal-3, for example, facilitates neutrophil phagocytosis of *Streptococcus pneumoniae* (60). Gal-9 can also bind and enhance the phagocytosis of *Pseudomonas aeruginosa* by neutrophils. While galectins have been shown to bind a variety of different bacterial species (51, 111), it is not clear whether this reflects a general phenomenon of galectin-mediated microbial clearance or only occurs following engagement of select microbial strains with unique glycan signatures. Regardless, in the setting of Gal-9, Tim3 appears to be involved (69). In addition to engaging bacterial pathogens, Gal-3 can also facilitate the phagocytosis of *Candida parapsilosis* yeast and *Candida albicans* hyphae, but not *C. albicans* yeast (112). However, Gal-3 may also directly kill *C. albicans* yeast (113). Gal-3 is secreted by neutrophils following exposure to yeast mannans, suggesting a mechanism whereby fungal exposure may trigger Gal-3-mediated removal (112). Gal-3 may also facilitate neutrophil phagocytosis of non-pathogens, such as red blood cells (114).

Galectins may also regulate immunity by inducing alterations in neutrophil function that directly and indirectly impact the immune activity of other cells. For example, neutrophils appear to perform helper activity through enhancing B cell antibody production, a process that requires Gal-3 (115). Neutrophils also produce more IL-17 in Gal-3 KO mice, suggesting that in addition to Gal-3 regulating dendritic cell IL-23, IL1β and TGFβ1 production, the reduction in *Histoplasma capsulatum* infection observed in Gal-3 KO mice may reflect an enhanced neutrophil-mediated Th17 response (116). Gal-9 can modulate neutrophil prostaglandin E2 production, which in turn reduces pro-inflammatory cytokine secretion by macrophages (117). In contrast to directly signaling cytokine responses in neutrophils, crystal forms of galectin-10, originally known as Charcot-Leyden crystal, can drive IL-1β production in macrophages when phagocytosed, which appears to result in neutrophil accumulation (118).

## Galectin Regulation of Neutrophil Turnover

**Figure 2:**
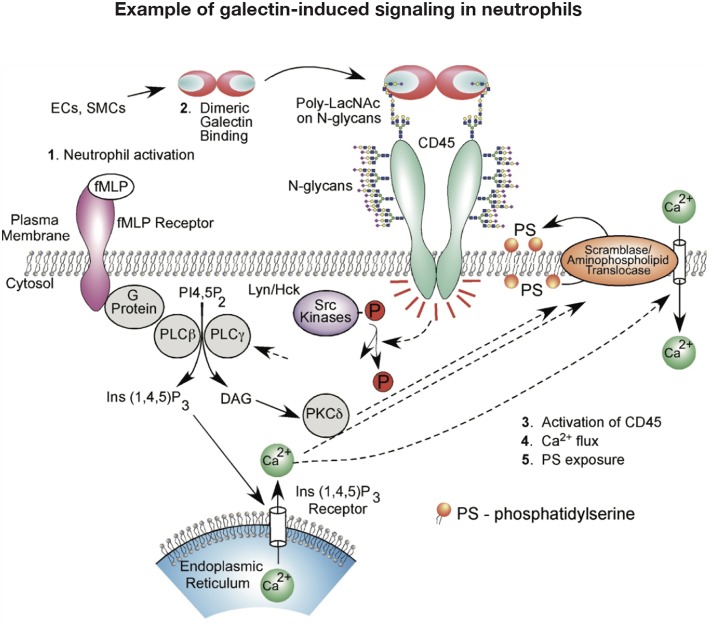
Example of galectin signaling in neutrophils. Galectin engagement of glycoconjugates on the cell surface can result in signaling events that alter neutrophil function. This schematic highlights potential signaling pathways engaged following galectin-1 binding that may result in phosphatidylserine (PS) exposure and subsequent neutrophil removal.

While galectins are differentially regulated in models of neutrophil development (119, 120), galectins may also govern neutrophil turnover. Given the role of galectins in regulating T cell viability, early studies similarly evaluated the potential role of galectins on neutrophil turnover. These initial studies investigated the effect of Gal-1 on neutrophils and promyelocytic HL-60 cells viability using Annexin V detection of phosphatidylserine (PS) exposure at the cell surface as a marker of apoptosis (121). Similar to T cells, Gal-1 signaled PS exposure in neutrophils. However, unlike cells undergoing apoptosis, Gal-1-induced PS exposure in neutrophils and HL60 cells occurred in the conspicuous absence of common features of apoptosis, including DNA fragmentation, cytochrome C release, mitochondrial potential changes or caspase activation (121–125). Despite the inability of Gal-1 to induce apoptosis in neutrophils, these cells remained sensitive to phagocytosis by macrophages (121), suggesting that Gal-1 possesses the unique ability to trigger neutrophil removal independent of cell death. Intriguingly, subsequent studies showed that this effect extended to other galectins, notably Gal-2, Gal-3, and Gal-4, which likewise stimulate PS exposure without concatenate apoptosis (124). However, it should be noted that pathways induced by at least Gal-1, Gal-2, and Gal-4 appeared to differ. While Gal-1 and Gal-2 induced an initial intracellular Ca^2+^ flux required for Gal-1-mediated PS exposure, a similar Ca^2+^ flux following exposure to Gal-4 is not observed (122, 124) (Figure 2). It should be noted that there have been conflicting results regarding the consequence of Gal-1 on neutrophil turnover. Additional studies demonstrated that under certain conditions Gal-1 may actually induce neutrophil apoptosis (114), while Gal-3 may indeed delay apoptosis (60). Differences in neutrophil sensitivity to assay conditions may in part account for these differences (126–128).

In addition to directly regulating neutrophil viability and turnover, galectins may also facilitate neutrophil clearance by macrophages. Recombinant Gal-3 enhances macrophage removal of apoptotic neutrophils (129), while Gal-3 KO macrophages have an impaired ability to phagocytose apoptotic neutrophils (60). Impaired Gal-3-mediated removal of neutrophils has also been attributed to worsening of the disease pathogenesis in asthma (130, 131). Gal-9 also co-localizes with corpses of neutrophils following NETosis, suggesting a potential role in the clearance of neutrophils following NETosis induction (132).

## Galectin Neutrophil Ligands

Definitive functional receptors for specific galectin signaling events in neutrophils have largely remained elusive (more than one may likely be involved), though studies strongly indicate CD66a and CD66b are at least in part responsible for ROS induction by Gal-3 (133, 134). IgM-mediated crosslinking of CD66b also induces IL-8 secretion, similar to Gal-3, suggesting that this indeed may be a functional ligand for Gal-3 (135). Despite similarities in ROS induction and overall neutrophil priming requirements for Gal-1 and Gal-3, early studies suggested that different receptors are engaged by Gal-1 and Gal-3 to induce these downstream events (64). The ability of blocking antibodies to CD43, but not CD45RO (another putative Gal-1 ligand) to inhibit Gal-1-induced neutrophil chemotaxis (98) corroborates the notion that Gal-1 may signal neutrophils through a different receptor. Although, it is not known whether CD43 also mediates Gal-1-induced ROS production. In contrast, αM integrin serves as receptor for Gal-8 induced adhesion of neutrophil to tissue culture plates (67), while Tim3 mediates Gal-9 enhancement of neutrophil microbial killing (69), suggesting that a variety of distinct neutrophil receptors may be engaged by different family members.

While many studies have defined galectin counter receptors on the surface of other immune cells, such as CD43 and CD45 on T cells, as the repertoire of glycosyltransferases can fundamentally differ between cell populations, these glycoproteins may or may not be decorated with suitable galectin ligands when expressed on neutrophils (136). As a result, several studies have instead focused primarily on the glycan ligands that support galectin-mediated signaling events in neutrophils (137). For example, several studies demonstrated that Gal-1, Gal-2, Gal-3 and Gal-8 prefer polylactosamine (polyLacNAc) ligands on the surface of HL60 cells. However, the mode of galectin interaction with polyLacNAc HL60 glycan recognition appears to fundamental differ. While Gal-3 and Gal-8 appear to prefer internal LacNAc glycan motifs within a polyLacNAc structure, Gal-1 and Gal-2 preferentially recognize the terminal LacNAc structure (138, 139). These differences have consequences on the sensitivity of HL60 cells to galectin signaling. While sialylation has little effect on Gal-3 binding or signaling of PS exposure, given the preference of Gal-1 and Gal-2 for the terminal LacNAc motif, sialylation can differentially impact Gal-1 and Gal-2 binding. Gal-2 fails to recognize any sialylated polyLacNAc structures, while Gal-1 binding appears to be preferentially inhibited by α2-6, but not α2-3 sialylation (138). Gal-8 glycan recognition is very different than Gal-1, Gal-2, and Gal-3. Unlike Gal-1, Gal-2, and Gal-3, Gal-8 is a tandem repeat galectin with two distinct carbohydrate binding domains. Gal-8 appears to dimerize through association with the N-terminal domain, while C-terminal domain engagement of polyLacNAc structures through internal LacNAc recognition is entirely responsible for Gal-8-induced PS exposure (139). Thus, while galectins can induce PS exposure in HL60 cells, the key features responsible for ligand engagement can differ. Whether similar glycan binding preferences dictate the ability of galectins to modulate neutrophil extravasation, chemotaxis and overall activation remains to be determined.

It should be noted that while a given glycoprotein or glycolipid may serve as the functional receptor for a galectin or several galectin family members, it is certainly possible that galectins signal neutrophils through clustering of several similarly glycosylated receptors to ultimately induce a particular signaling outcome. Consistent with this possibility, Gal-3 clusters neutrophil counter receptors alone and in the context of adhesion to endothelial cells (140). LPS enhances oligomerization of Gal-3 (50), augmenting the ability of Gal-3 to signal neutrophil activation and possibly contributing to the increased lethality observed when Gal-3 is injected intraperitoneally (IP) with LPS when compared to Gal-3 alone (50).

## Redox as a Regulator of Galectin Biology

Given the ability of galectins to broadly influence neutrophils, and in sometimes opposing manners, one critical challenge to the field is in understanding the dynamics of the regulatory network controlling galectin-glycan interactions to allow for proper control of neutrophil function. Clearly one key element is the expression of glycans on cognate receptors within the extracellular space. However, one often overlooked mechanism may be in the rich and fluctuating redox environment often accompanying inflammation, inflammatory resolution and eventual tissue repair.

Protein oxidation is one of the strongest regulatory modifications linked to galectins, and is also one of the first identified. In fact, the identification of galectins remained elusive until it was discovered that their purification required reducing conditions (24, 26). In these studies, the authors found that tryptophan oxidation not only inactivated the ability of electrolectin to bind to lactose, but also that this inhibitory effect was influenced by binding to cognate glycans themselves, as pre-incubation with lactose prevented redox-dependent inactivation. Oxidation also occurred in a pH sensitive manner, with optimal yields only occurring in the setting of a neutral pH. Thus, in the setting of acute injury, which often has high acid and oxidant loads, galectin activity may be titrated toward high affinity interactions.

**Figure 3:**
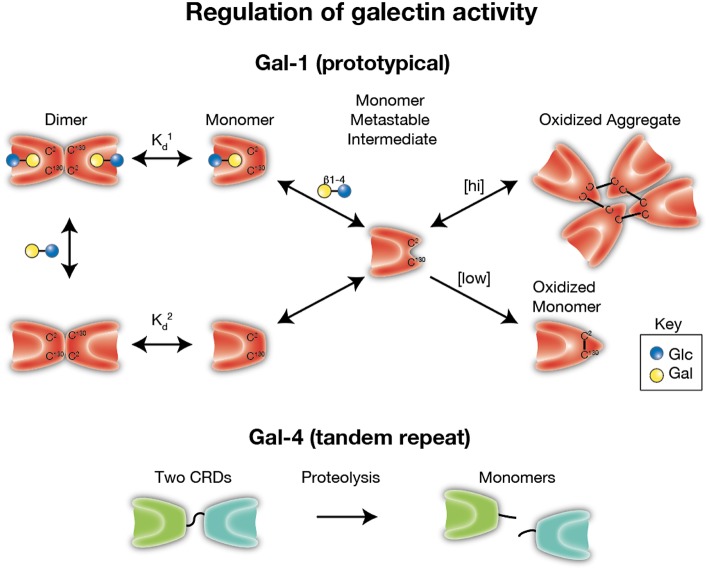
Regulation of galectin activity. Several galectins require reduced thiol groups to maintain carbohydrate recognition activity. Formation of intra- and inter-molecular disulfide bridges can result in significant conformational changes the preclude carbohydrate recognition. As monomers appear to be a key intermediate in oxidative inactivation and carbohydrates can drive dimerization, ligand appears to reduce oxidative inactivation by facilitating dimer formation (${\text{K}}_{\text{d}}^{1}$ < ${\text{K}}_{\text{d}}^{2}$). [hi] = higher concentrations of galectin-1 (Gal-1). [low] = lower concentrations of Gal-1. In contrast, several galectins, especially tandem repeat and chimeric galectins, rely on linker peptide bound carbohydrate recognition domains or N terminal collagen-like domains to facilitate dimerization. Cleavage of intervening peptides that connect oligomerization domains to functional carbohydrate recognition domains can render carbohydrate recognition domains monomeric and therefore incapable of generating signaling lattices typically thought to be required for optimal galectin-mediated signaling.

Subsequent studies performed on vertebrate galectins, in particular Gal-1, have similarly observed sensitivity to oxidation, however these studies found that redox dependent control of Gal-1 appears to impinge on the modification of critical cysteine residues present on the Gal-1 backbone (27, 30–33, 141). As with redox-driven inactivation of other systems, oxidation appears to promote disulfide bond formation (notably on Cys2, Cys16, and Cys88) leading to intramolecular interactions which disrupt the ability of the carbohydrate binding domain to recognize and bind to cognate glycans (33, 142–145) (Figure 3). This effect appears to be completely blocked by alkylation and site directed mutagenesis of these cysteine residues also results in stable proteins with sustained binding activity (27, 30). Intriguingly, as was originally observed with electrolectin, redox-driven inactivation of Gal-1 appears to be regulated by the presence of ligand; whereas free Gal-1 monomer is relatively quickly inactivated, ligand-binding by Gal-1, which induces dimerization, increases resistance to inactivation (126, 146, 147). Similar findings have now been documented with Gal-2, another prototypical galectin, where oxidation of Cys57 appears to result in its oligerimization and subsequent inactivation, an effect which can be abrogated by endogenous nitric oxidase in the gastrointestinal tract (148, 149). However, whether this type of regulation occurs with other galectins, or if this affects the ability of galectins to mediate carbohydrate-independent interactions, remains incompletely understood. Intriguingly, though oxidation appears to clearly disrupt Gal-1 glycan binding activity and subsequent dimerization, in certain settings oxidation alters Gal-1 biological function in a manner that appears to be independent from its lectin properties. This observation stems from studies looking at a unique role of Gal-1 in promoting axonal regeneration of peripheral nerves where it was observed that while ectopic oxidized Gal-1 could enhance the rate of axonal growth from transected dorsal root ganglia, alkylated Gal-1 (which prevents redox-dependent conformational changes) could not (33, 150). These results strongly suggest a role for oxidized Gal-1 in tissue regeneration (33, 151–157). Subsequent studies revealed that Gal-1 was not only expressed but secreted from regenerating nerves, and that neutralizing antibodies against Gal-1 could strongly inhibit axon regeneration *in vivo* (158). Studies have now revealed that oxidized Gal-1 can stimulate macrophages to initiate regenerative responses during axonal injury (156, 159), and through this pathway primes the system for repair.

All these data suggest that Gal-1 appears to act as a morpheein (160), a secreted factor which under distinct conformational conditions adopts certain biologic behaviors; whereas reduced Gal-1 maintains its lectin-binding immune-modulatory activity; cysteine oxidized Gal-1 adopts a new behavior with a tailored regenerative response. The degree to which this effect can be observed with other galectins, including other prototypical galectins remains unknown. Moreover, whether the effect remains specific to regenerating axons, or whether oxidized Gal-1 can stimulate macrophages to promote restitution in other tissues is similarly unknown (Figure 3).

## Proteolytic Regulation of Galectins

While the extracellular environment during acute inflammatory responses is rich in reactive oxygen species and electrophiles which could exert regulatory influences on galectins (as detailed above), it is also well-known that several proteolytic enzymes (such as matrix metalloproteinases or MMPs) are elevated during this time which could also serve to regulate galectin activity. Early studies showed that both MMP-2 (gelatinase-A) and MMP-9 (gelatinase-B) can cleave Gal-3 at specific residues within its N-terminal tail (Ala^62^-Tyr^63^), leading to reduced cell surface expression in human breast cancer cell lines (161). Subsequent studies confirmed these findings *in vivo* (162), and showed that while this cleavage led to reduced N-terminal self-association/oligomerization of Gal-3, its ability to bind glycans was enhanced (163). Thus, as with oxidation, cleavage appears to act as a switch on galectin function, where certain functions that rely on N-terminal oligomerization (e.g., hemagglutination) are reduced, while others that do not require this function are potentially enhanced.

Regulation of galectin activity by proteases does not appear to be limited to gelatinases (e.g., MMP-2 and - 9); a variety of other MMPs have been implicated in altering Gal-3 expression/activity. MMP-7 (i.e., Matrilysin-1), which is expressed in inflamed tissues often at the leading edge of gastrointestinal ulcers, was shown to cleave Gal-3 at three separate sites (including Asp^72^-Tyr^73^) and inhibit Gal-3 driven wound healing activity in T84 cells (162). MMP-13 (i.e., collagenase-3) was shown to cleave Gal-3 at sites identical to MMP-2 and MMP-9. This action was correlated with altered expression in chondrocytes (164). MMT-MMP (Membrane type 1 matrix metalloproteinase) was similarly found to enhance Gal-3 cleavage, though this effect was presumed to occur through indirect activation of MMP-2 and MMP-9 (165).

In addition to the matrix metalloproteinases, several other classes of proteinases have been implicated in galectin regulation. The serine peptidase PSA (protate specific antigen) was found to cleave Gal-3 in seminal plasma at Tyr^107^-Gly^108^ and result in a functional monovalent lectin (166), akin to what has been observed with MMPs. Neutrophil elastase can likewise cleave Gal-3 (59). Regulation of galectin cleavage is not limited to endogenous proteases, as *Staphylococcus aureus* and *Leishmania major* possess similar proteolytic activity toward Gal-3 (167, 168). Recent studies have shown that Gal-8 and Gal-9 are susceptible to cleavage by the serine protease thrombin (68). This effect appears to be specific to Gal-8 and Gal-9, as thrombin susceptibility was not observed in galectin-1, -2, -3, -4, -7, -10, and -13. Intriguingly, Gal-8 and Gal-9 cleavage only occurred in long isoforms of these proteins, as short and medium isoforms were either resistant or lacked the site required for cleavage. In both instances, thrombin mediated cleavage abrogated the ability of the long isoform of Gal-8 (Gal-8L) to mediate neutrophil adhesion and Gal-9 eosinophil-chemoattractant activity, respectively. Thus, in the setting of acute inflammatory responses and tissue injury, which are often accompanied by an influx of coagulation proteins including thrombin activation and other proteases (169), this mode of regulation may serve as an additional means to curb galectin activity and prevent excessive tissue damage from inappropriate inflammatory cell activation.

## Bringing It Together: Galectins as Unique Regulators of Overall Neutrophil Function

**Figure 4:**
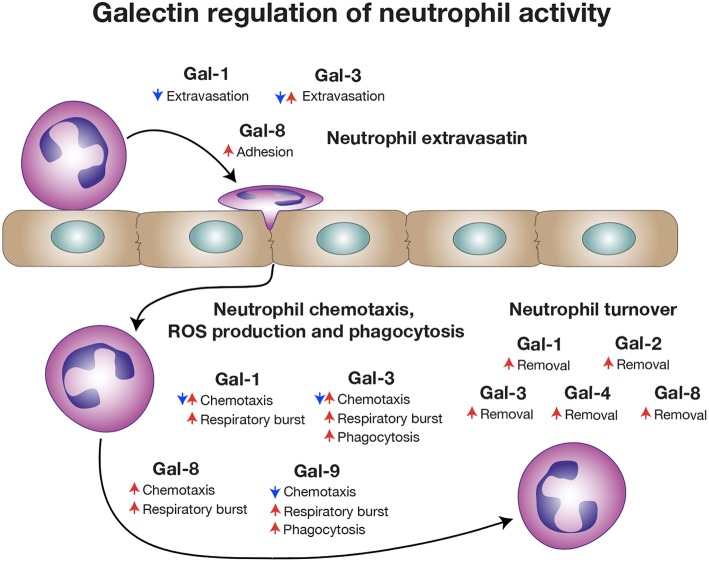
Galectins regulate a broad range of neutrophil activities. Different galectin family members influence various stages of neutrophil biology, ranging from extravasation, activation and chemotaxis to eventual turnover. Some galectins have been reported to have opposite activities on neutrophils that may reflect different types of inflammation.

The distinct localization of galectins, their ability to selectively bind cell surface carbohydrates and their sensitivity to oxidative inactivation and proteolytic cleavage likely provided a unique evolutionary substrate to regulate the temporal and spatial activity of neutrophils during inflammation. Galectin expression within vascular endothelial cells and possibly in neutrophils themselves may contribute to extravasation, early activation and even chemotaxis (170). However, unlike most immune regulators, which are either synthesized and released following pathogen exposure, injury and/or selectively expressed by distinct immune cells (171), many galectins are found at high levels in a variety of tissues under baseline conditions (172). Thus, while galectins may interact with neutrophils intravascularly and therefore regulate early events involved in neutrophil extravasation, the expression of galectins in a variety of tissues provides additional opportunities for galectins to regulate neutrophil function (Figure 4).

The broad tissue distribution of galectins, coupled with their unique sensitivity to oxidative inactivation and proteolytic cleavage, may provide some insight into the temporal and spatial regulation of neutrophil function. Unlike most cytokines and chemokines, the vast majority of galectins reside in the cytosol, consistent with their lack of a signal peptide and consequential translation on free ribosomes (173). Following tissue injury, total levels of galectin can be upregulated, signaling an active production of these proteins (174). However, various forms of injury can also result in the release of galectins into the extracellular space, a process that may reflect active secretion, but also is likely a consequence of direct cellular injury (174). Initial release from cells requires galectins to transition from a relatively reducing environment largely devoid of proteases that target galectin function, into an environment that is oxidative in nature where proteases abound. While engagement of carbohydrate ligand can inhibit galectin oxidation, saturation of available ligands, coupled with proteolytic cleavage, may render most galectins inactive immediately following an injury event. This relatively rapid loss of galectin activity may aid the inflammatory response by preventing galectins from inhibiting productive chemotaxis and prematurely inducing neutrophil turnover. Furthermore, as significant galectin accumulation in the intravascular space can result in platelet activation, leukocyte aggregation and vascular stasis (175), spatial regulation of galectin activity may also be important in preventing galectin-induced vascular blockage, which would be expected to increase tissue ischemia and prevent additional leukocyte recruitment (176).

As neutrophils effectively remove pathogens and necrotic tissue in the settings of inflammation, these cells can also infringe on surrounding viable tissue (2, 3). In contrast to T cells, NK cells and other immune effectors, once activated, neutrophils do not process clear receptors capable of demarcating self from non-self, especially in the absence of pathogen specific antibodies. As a result, activated neutrophils can cause significant damage to viable tissue (2, 3). Indeed, inappropriate neutrophil activation not only exacerbates inflammatory responses in general, but also underlies the pathogenesis of a variety of disease states (3, 7–11). Galectins may provide some spatial control for neutrophils. As neutrophils encroach on and damage viable tissue surrounding the area of initial injury, intracellular stores of reduced, intact and therefore active galectin are released. Galectin engagement of neutrophils in these peripheral areas may therefore serve to reduce chemotaxis and enhance neutrophil removal (63, 114, 121, 122, 124). This spatial and temporal regulation of galectin activity and consequently neutrophil function may be important in limiting neutrophil-mediated injury while also inducing neutrophil turnover. Galectin-induced ROS production may therefore not only reflect an important early activator of neutrophil function and microbiocidal activity, but may also facilitate complete microbial killing before galectin signaling programs finalize events that mark neutrophils for removal. The ability of recombinant galectin to enhance tissue repair in some models may, in part, reflect the ability of galectins to favorably regulate leukocyte turnover in the setting of ongoing tissue injury and inflammation (177, 178).

**Figure 5:**
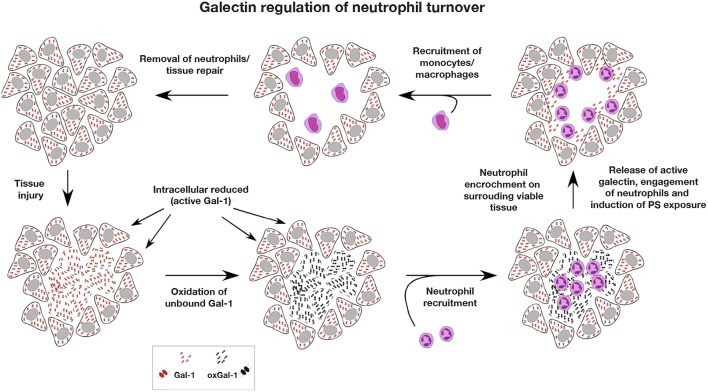
Galectin regulation of neutrophil turnover. As the intracellular environment is reducing, intracellular stores of galectin remain active. However, following cellular injury, intracellular galectin becomes exposed to the extracellular oxidizing environment, where galectin oxidation and inactivation may occur. Rapid infiltration of neutrophils following injury allows for neutralization of potential pathogens and removal of necrotic tissue. As most extracellular galectin may become oxidized following injury prior to significant neutrophil recruitment, the ability of galectins to induce neutrophil turnover would be compromised, preventing galectins from inhibiting a productive inflammatory response. Following removal of necrotic tissue and pathogens, neutrophil encroachment on surrounding viable tissue results in cellular damage and release of reduced and therefore active galectin. Released galectins then engage neutrophils impinging on surrounding viable tissue, induce an oxidative burst that facilitates killing of ingested pathogens and the induction of PS exposure. As galectin-induced PS exposure occurs in the absence of apoptosis, this allows neutrophils to maintain membrane integrity in an otherwise inflammatory environment until successfully phagocytosed by monocyte-differentiated macrophages, which are typically outnumbered by neutrophils and are recruited after significant neutrophil influx. Once neutrophils are removed and inflammation subsides, tissue repair and regeneration ensue.

While there are a variety of distinct forms of programmed cell death, ranging from apoptosis to necroptosis (179), the ability of galectins to induce PS exposure in the absence of cell death represents a distinct cell removal mechanism that may have uniquely evolved to eliminate neutrophils and perhaps other innate immune cells. As apoptosis typically occurs to prevent inflammation, the ability of galectins to induce PS exposure in the absence of apoptosis may allow neutrophils to maintain membrane integrity in a highly inflammatory and membrane damaging environment until successfully phagocytosed. This is especially important when considering that once neutrophil undergo apoptosis, there is a short window of time before late apoptosis, which is signified by loss of membrane integrity, occurs (180). If apoptotic neutrophils are not quickly phagocytosed, late apoptosis would be predicted to result in unregulated release of neutrophil contents, causing further tissue injury, additional inflammation and impaired inflammatory resolution. Furthermore, as the number of neutrophils often far outweighs the number of macrophages responsible for their removal (3), the ability of galectins to flag neutrophils for removal without inducing actual apoptosis, may likewise allow neutrophils to maintain membrane integrity while awaiting removal. While the residue of galectin released from injured tissue that is not bound to neutrophils, the ECM or other ligands would be predicted to undergo oxidative and/or proteolytic inactivation (126, 147), given the ability of at least oxidized Gal-1 to induce tissue regeneration, oxidized galectin, perhaps in the presence of other tissue factors, such as resolvins (181), may then be uniquely poised to begin the signals necessary for tissue repair as resolution of the inflammatory response occurs (Figure 5).

## Future Directions: Challenges and Opportunities in Harnessing Galectin Regulation of Neutrophil Function

The ability of galectins to regulate neutrophil function suggests that these proteins may serve as useful pharmacological agents to favorably alter disease states marked by inadequate or exuberant neutrophil function. Consistent with this possibility, the earliest description of galectin-mediated immune regulation occurred following the exogenous delivery of electrolectin in a model of myasthenia gravis. Intriguingly, while the initial hypothesis was that delivery of electrolectin would stabilize the neuromuscular junction, additional experiments demonstrated that electrolectin actually inhibited the immune response required to induce myasthenia gravis (182–184). These results not only provided the first evidence that galectins may regulate immunity, but also suggested that galectin family members may serve as useful pharmacological agents to favorably alter immune function.

Subsequent studies demonstrated improved outcomes could also be achieved following galectin injection in additional models of immune-related pathology, including Concanavalin-A-induced hepatitis, collagen-induced arthritis, experimental autoimmune uveitis and experimental autoimmune encephalitis (185–187). These collective studies, which primarily focused on the outcome of galectin-1 injection, suggested that galectins can inhibit immune-related pathology by reducing the pro-inflammatory activities of macrophages, DCs and T cells (16–19, 185–188). While most of these early studies did not directly examine the impact of galectin injection on neutrophil numbers and function, subsequent studies suggested that recombinant galectin can inhibit neutrophil extravasation, chemotaxis and overall activation (63, 72–78, 82–85, 88, 98, 99, 101–103). Taken together, these results suggest that harnessing the ability of galectins to regulate neutrophil function may have therapeutic potential.

The vast majority of studies that have examined the impact of exogenous delivery of galectin on immune function have employed an intraperitoneal (IP) delivery route, a common approach of introducing substances in small rodent models, but one that is seldomly employed clinically (72, 189). The ability of galectins to bind common glycan motifs present on nearly every cell type is very different from the binding specificity of most naturally occurring or synthetic molecules designed to target immune function. Engagement of glycoconjugates in solution or on the surface of cells within the peritoneal cavity would be predicted to impact the overall biodistribution of galectin following IP delivery. Indeed, it is not clear that galectin injected IP actually arrives at the location of injury, inflammation or immunomodulation. The impact of galectin injection on neutrophil function may therefore reflect indirect effects of galectins that result from general immunosuppression or other types of immunomodulation. As previous studies have suggested that galectins can regulate nearly every immune cell studied (in addition to their ability to alter the activity of many non-immune cells) (16–19, 190), the outcome of galectin injection may reflect a pleotropic effect, where galectins induce alterations in the activities of other cells that converge to influence neutrophil function.

Injection of galectins intravenously (IV) would appear to be a more favorable approach to avoid engagement of intraperitoneal contents and possibly model clinical routes of delivery more accurately. However, unpublished work by numerous labs has demonstrated that IV injection of active galectin-1 results in rapid death, presumably due to immediate galectin-induced hemagglutination and vascular stasis. While galectins have been reported to circulate, galectins detected as serum biomarkers of heart disease and other conditions likely represent inactive galectin as the assays employed in these studies utilize methods of antigen detection that do not directly assess galectin activity (191). As previously discussed, the sensitivity of galectin to oxidation likely provides critical spatial and temporal regulation that reduces the probability of galectin-mediated vascular stasis observed following an IV bolus of galectin. As the bivalent properties of galectins are not only thought to be responsible for crosslinking counter receptors on the neutrophil surface, but also contribute to hemagglutination, separating the intrinsic biophysical features of galectins that contribute to hemagglutination from their biological activities will likely be difficult (121).

Given the potential challenges of using galectins as modulators of neutrophil function clinically, alternative approaches may be required to fully harness the therapeutic potential of galectins to modify neutrophil function. Several reports have described various synthetic analogs of galectin ligands that appear to specifically inhibit distinct galectin family members (192, 193), providing a potential opportunity to reduce galectin-mediated activation of neutrophils in settings where excessive neutrophil activity may be unfavorable. However, in order to augment a galectin-mediated neutrophil outcome without using recombinant galectin, the actual receptors responsible for mediating the effects of galectins will likely need to be identified and targeted. Identifying galectin ligands on neutrophils that mediate distinct aspects of galectin-dependent regulation not only holds promise in avoiding some of the challenges associated with galectin delivery, but may also provide a more specific approach to dissect different aspects of galectin neutrophil regulation and therefore more deliberately modify neutrophil function in the setting of infection, inflammation or injury. Such an approach may employ antibodies that target protein or glycoprotein epitopes specific to the target receptor, thereby avoiding the potential pleotropic effects that can occur following galectin engagement of more common glycan ligands. However, if the signaling outcome of galectins reflects engagement and clustering of multiple receptors, it may be difficult to recapitulate these activities using a single antibody-based or similar surrogate approach.

Regardless of whether identifying and targeting galectin receptors will provide a suitable substitute for recombinant galectins as a therapeutic strategy, defining functional counter receptors for galectins will allow additional approaches to be used when seeking to further define the roles of galectins on neutrophil activity *in vivo*. As different galectins appear to regulate neutrophil function through similar pathways (16–19), genetic approaches utilizing galectin KOs can be deceiving when only negative results are obtained. As *in vivo* studies have often been driven by initial *in vitro* observations and early *in vitro* data suggest that multiple galectins possess the ability to modulate neutrophil behavior in a similar manner, significant functional redundancy between different galectin family members *in vivo* may reduce the likelihood that a clear phenotype will be observed when using recipients deleted of only one or even several galectin family members. Furthermore, as galectins also regulate other immune populations (16–19), when immunological outcomes are observed following genetic deletion of galectins, it can be difficult to interpret these results, as a particular phenotype observed in a galectin KO may reflect galectin regulation of neutrophil function and/or an indirect outcome of galectin activity on a variety of cell populations, which may in turn influence neutrophil function. Although floxed KO alleles have not be described for any galectin, even this approach, wherein individual galectins can be specifically deleted in neutrophils, may likewise inadequately address this issue, as galectins are expressed by many different cell types, making it virtually impossible to prevent at least extracellular galectin from engaging neutrophils and potentially altering their function. Defining functional galectin receptors on neutrophils will therefore provide additional genetic targets that can be specifically deleted on neutrophils and thus allow an important complementary approach when seeking to examine the potential impact of various galectin family members on neutrophil function *in vivo*.

## Conclusion

Studies over several decades demonstrate that galectins can regulate a wide variety of neutrophil functions. The ability of galectins to bind a broad range of receptors and similarly be regulated by unique oxidative and proteolytic processes, suggests that evolution selected these unique immune regulators to temporally and spatially shape neutrophil function. In doing so, galectins appear to serve as critical regulators of neutrophil biology. While many *in vivo* studies appear to corroborate galectin activity on neutrophil function, additional studies are needed to formally test many of these hypotheses *in vivo*.

## Author Contributions

All authors listed have made a substantial, direct and intellectual contribution to the work, and approved it for publication.

### Conflict of Interest Statement

The authors declare that the research was conducted in the absence of any commercial or financial relationships that could be construed as a potential conflict of interest.
